# Growth Factor Stimulation Improves the Structure and Properties of Scaffold-Free Engineered Auricular Cartilage Constructs

**DOI:** 10.1371/journal.pone.0105170

**Published:** 2014-08-15

**Authors:** Renata G. Rosa, Paulo P. Joazeiro, Juares Bianco, Manuela Kunz, Joanna F. Weber, Stephen D. Waldman

**Affiliations:** 1 Human Mobility Research Centre, Kingston General Hospital and Queen's University, Kingston, Canada; 2 Department of Histology and Embryology, Institute of Biology, University of Campinas (UNICAMP), Campinas, Brazil; 3 School of Computing, Queen's University, Kingston, Canada; 4 Department of Mechanical & Materials Engineering, Queen's University, Kingston, Canada; 5 Department of Chemical Engineering, Ryerson University, Toronto, Canada; 6 Keenan Research Centre of the Li Ka Shing Knowledge Institute, St. Michael's Hospital, Toronto, Canada; Texas A&M University Baylor College of Dentistry, United States of America

## Abstract

The reconstruction of the external ear to correct congenital deformities or repair following trauma remains a significant challenge in reconstructive surgery. Previously, we have developed a novel approach to create scaffold-free, tissue engineering elastic cartilage constructs directly from a small population of donor cells. Although the developed constructs appeared to adopt the structural appearance of native auricular cartilage, the constructs displayed limited expression and poor localization of elastin. In the present study, the effect of growth factor supplementation (insulin, IGF-1, or TGF-β1) was investigated to stimulate elastogenesis as well as to improve overall tissue formation. Using rabbit auricular chondrocytes, bioreactor-cultivated constructs supplemented with either insulin or IGF-1 displayed increased deposition of cartilaginous ECM, improved mechanical properties, and thicknesses comparable to native auricular cartilage after 4 weeks of growth. Similarly, growth factor supplementation resulted in increased expression and improved localization of elastin, primarily restricted within the cartilaginous region of the tissue construct. Additional studies were conducted to determine whether scaffold-free engineered auricular cartilage constructs could be developed in the 3D shape of the external ear. Isolated auricular chondrocytes were grown in rapid-prototyped tissue culture molds with additional insulin or IGF-1 supplementation during bioreactor cultivation. Using this approach, the developed tissue constructs were flexible and had a 3D shape in very good agreement to the culture mold (average error <400 µm). While scaffold-free, engineered auricular cartilage constructs can be created with both the appropriate tissue structure and 3D shape of the external ear, future studies will be aimed assessing potential changes in construct shape and properties after subcutaneous implantation.

## Introduction

Total or partial reconstruction of the external ear remains one of the most difficult challenges in reconstructive surgery [1]–[4]. While there are a few approaches currently available (e.g. sculpted autologous costal cartilage grafts, alloplastic implants), these procedures carry significant morbidity, are prone to surgical complications, and the natural shape of the ear can be difficult to recreate [1], [3], [5]. To address these deficiencies, one promising method for external ear reconstruction involves the use of anatomically shaped, tissue engineered cartilage constructs. Despite this potential, several technical hurdles need to be overcome to develop functional constructs suitable for ear reconstruction [2].

One of the major shortcomings of this approach is that the native structure of auricular cartilage is not easily reproduced *in vitro*. Auricular cartilage is an elastic cartilaginous tissue that possesses with two discrete zones: (i) a central cartilaginous region (or chondrium) and (ii) an outer fibrous region, termed the perichondrium. The central, cartilaginous region is rich in collagen II and large aggregating proteoglycans as well as abundant elastic fibres (crosslinked elastin and associated microfibrils) [6]. The central cartilaginous region is surrounded by the perichondrium — a thin fibrous tissue layer believed to be essential for the growth and maintenance of elastic cartilage [1], [7], [8]. The perichondrium is rich in collagen type I fibres, small non-aggregating proteoglycans and contains numerous fibroblastic-like cells. Although various different types of biomaterial scaffolds have been explored for auricular cartilage engineering (e.g. polyglycolic acid (PGA), polycaprolactone (PCL), polylactic acid (PLA)) [1], [3], these scaffolds are primary aimed at recreating the cartilaginous zone even though the need for the engineered constructs to possess a functional perichondrium has long been noted [7], [9], [10]. Relatively few studies have been able to address this shortcoming with neo-perichondrial formation typically only observed after subcutaneous implantation [4], [11], [12]. While a significant advance, this necessitates the need for a pre-implantation stage (e.g. abdominal) to functionalize the construct prior to implantation at the defect site [4].

Recently, we have developed a novel approach to create large-sized, engineered auricular cartilage constructs directly from a very small population of donor cells (∼13,000 cells/cm^2^) without a separate cell expansion phase or scaffold [13]. The cells are cultivated in a continuous flow bioreactor which elicits the extensive growth of new cartilaginous tissue. The developed constructs adopt the structural appearance of native auricular cartilage with distinct cartilage and perichondrium regions [13] — suggesting that the development of procedures with fewer clinical stages can be achieved. However, elastin expression by auricular chondrocytes *in vitro* can be problematic [7], [14]–[16] with generally reduced expression in the engineered constructs [17]. Growth factor stimulation is a widely used method to improve tissue formation as well as the expression of specific extracellular matrix (ECM) macromolecules. In terms of elastogenesis, several studies have demonstrated improved elastic fibre formation in different cell types with supplementation of insulin, IGF-1, TGF-β1, or CFGF [18]–[21]. Interestingly, a subset of these growth factors has also been shown to elicit an anabolic response in cartilage; specifically, insulin, IGF-1, and TGF-β1 [22], [23]. As an attempt to improve the quality of the engineered auricular cartilage constructs, the purpose of this study was to investigate the potential of insulin, IGF-1, and TGF-β1 stimulation during bioreactor culture to improve the expression and localization of elastic cartilaginous ECM proteins in the engineered constructs in a similar fashion to native auricular cartilage.

## Materials and Methods

### Rabbit Auricular Chondrocyte Harvest and Isolation

This study was performed with approval from the University Animal Care Committee (UACC) at Queen's University. Full thickness auricular cartilage was harvested from the ear of adolescent female New Zealand white rabbits (∼500 mg of extracted tissue from 2±0.5 kg; ∼12 weeks old animals) (Charles River Laboratories, Wilmington, USA) to reflect the general age of patients undergoing auricular reconstruction. Ears were completely dissected free of skin with the inner chondrogenic layer and the outer perichondrial layer were kept entirely intact. The tissue was cut in small fragments and digested with 0.5% protease (w/v) (Sigma-Aldrich, Oakville, Canada) followed by 0.15% collagenase A (w/v) (Roche Diagnostics Canada, Laval, QC) in Ham's F12 media (Hyclone, Logan, USA) overnight at 37 °C with 95% relative humidity and 5% CO_2_. Auricular chondorcytes were isolated by centrifugation (700×g for 7 minutes) with the resultant cell pellet washed 3 times with Ham's F12 media. Viable cells, determined by Trypan Blue dye (Sigma-Aldrich) exclusion [24] were then seeded in low-density monolayers in a continuous flow bioreactor. To minimize inter-animal variability, tissue was obtained from several ears (up to 3 per experiment obtained from different animals) and pooled together. Tissue constructs were generated from two different batches of isolated cells (total of N = 4–6 animal donors). Native tissue samples were harvested from the same animals and were used for testing (histological, immunohistochemical, biochemical, and mechanical) immediately after harvest.

### Continuous Flow Bioreactor

A continuous flow bioreactor system was used to maintain a constant supply of fresh medium to the developing construct in a low fluid shear environment [25], [26]. Briefly, the reactor consisted of multichannel vented polypropylene chambers to house single constructs (3 cm^2^ containing a maximum media volume of 4 mL). A constant 10 µL/min flow of fresh media (from aerated reservoirs) was provided by a peristaltic pump (Ismatec, Cole Parmer Canada, Anjou, Canada) with waste media collected in a vented reservoir (resulting in an average residence time of 6.67 hours). The bioreactor was housed in an incubator maintained at 37°C with 95% relative humidity and 5% CO_2_.

### Hormone and Growth Factor Supplementation

To determine the most effective stimulatory factors to improve the accumulation of specific tissue constituents in the engineered elastic cartilage constructs, three different hormone/growth factors were investigated: 10 nM IGF-1 (Peprotech, Rocky Hill, NJ, USA), 100 nM Insulin (Sigma, St Louis, MO, USA), or 0.1 nM TGF- β1 (Peprotech, Rocky Hill,NJ, USA). The selected concentrations were based on the mid-range concentrations used in previous studies [18]–[21]. Isolated cells were seeded in low-density monolayers (13,000 cells/cm^2^ or 40,000 cells/well) directly on the bottom surface of the reactor wells in Ham's F12 media (Hyclone, Logan, USA) supplemented with 20 mM HEPES (4-2(2-hydroxyethyl) piperazine-1-ethanesulfonic acid) (Sigma-Aldrich), 14 mM sodium bicarbonate (NaHCO_3_), 100 µg/mL ascorbate, 20% FBS, and an antibiotic solution containing: 100 U/mL penicillin, 100 µg/mL streptomycin and 0.25 µg/mL amphotericin B (Sigma-Aldrich) in the presence, or absence, or additional hormone/growth factors (IGF-1, insulin or TGF- β1).

After seeding, all preparations were maintained under no-flow conditions for 48 hours. Preparations were then cultured under a constant media flow rate of 10 µL/min for a period of 4 weeks. Media reservoirs were changed every 2–3 days and supplemented with fresh hormone/growth factors, ascorbic acid and antibiotics. After the 4 week culture period, developed constructs were harvested, weighed (wet weight). Constructs were tested mechanically and then divided into three parts and processed for histological/immunohistochemical evaluation, biochemical analyses and transmission electron microscopy, respectively.

### Assessment of Tissue Thickness and Mechanical Properties

The thickness of the tissue constructs was determined using a needle probe method [27] in conjunction with a Mach-1 Micromechanical Testing system (Biomomentum, Laval, QC, Canada) equipped with a 1 kg load cell (0.05 g resolution) averaged over three random locations in the construct. The thickness of native tissue samples was measured using calipers (0.5 µm resolution). Mechanical testing of native auricular cartilage and engineered cartilage constructs was then performed using a Mach-1 Micromechanical Testing system (Biomomentum, Laval, QC, Canada) equipped with a 1 kg load cell. Tissue mechanical properties (elastic modulus and Poisson's ratio at 37°C in Ham's F12 media) was determined using a double compressive indentation method [25], [28], [29] using two plane-ended indentors (2 and 6 mm diameter). Compressive indentations were conducted at a ramp rate of 10% strain/s to a maximum of 10% strain. Samples were then allowed to equilibrate in media for approximately 20 minutes between indentations. The resulting force-deformation response from both indentations (collected a frequency of 10 Hz) were then used to determine the elastic modulus and Poisson's ratio of the tissue sample using custom-designed code based on the theoretical model of cartilage indentation [28], [29].

### Histological Evaluation

Engineered constructs as well as native auricular cartilage tissue samples were fixed in 4% paraformaldehyde (in 0.1 M PBS, pH 7.2) for 24 hours, dehydrated in graded ethanol solutions and embedded in paraffin at 65°C. Thin (5 µm thick) sections were cut and mounted on Superfrost slides (Fisher Scientific, Mississauga, Canada) and dried for 24 hours at 37°C. Sections were stained with safranin-O (proteoglycan stain), Weigert's resorcin-fuchsin (elastin/elastic fiber stain) or hematoxylin & eosin (H&E; general connective tissue stain). Stained sections were examined by light microscopy using a Zeiss Axio-Image M1 microscope (Göttingen, Germany).

### Immunochemical Localization of Collagen Types and Elastin

Immunohistochemical localization of collagen types I and II in the engineered constructs and native auricular cartilage tissues samples was performed as previously described [21], [25]. Briefly, after deparaffinization and dehydration, sections were enzymatically treated with 0.05% of trypsin (pH 7.8) for 30 minutes at 37°C to facilitate antibody binding. Endogenous peroxidase activity was blocked with 1% H_2_O_2_ and 1% BSA (in PBS) for 30 minutes. Sections were then incubated with mouse monoclonal antibodies against collagen type I (ab90395 1∶100; USA) or collagen type II (II-II6B3 at 187 µg/mL; Developmental Studies Hybridoma Bank, Iowa, USA) all diluted in 1% BSA (in PBS, pH 7.4) overnight at 4°C. Following primary antibody incubation, sections were rinsed in PBS (pH 7.4), and incubated with biotinylated anti-mouse secondary antibodies (Vector Laboratories Inc., Burlingame, USA) using the Vectastain Elite ABC kit (Vector Laboratories) for 2 hours at room temperature, followed by incubation with diaminobenzidine (DAB) for 6 minutes at room temperature. The sections were counter-stained with Harris' hematoxylin and mounted in permanent mounting medium. Stained sections were examined by light microscopy using a Zeiss Axio-Image M1 microscope (Göttingen, Germany).

Immunofluorescence localization of elastin and collagen X in the engineered constructs and native auricular cartilage tissues samples was also performed as previously described [21], [25]. Briefly, after deparaffinization and dehydration, sections were enzymatically treated with 0.25 units/mL chondroitinase ABC (Sigma-Aldrich) in tris-acetate buffer (40 mM tris acetate with 1 mM EDTA, pH 8.5) for 1 hour at 37°C followed by 0.25 units/mL keratinase (Sigma-Aldrich) tris-acetate buffer for 30 minutes at 37°C to facilitate antibody binding. To reduce non-specific protein binding, the sections were blocked with 1% BSA (in PBS) for 30 minutes at room temperature. Sections were then incubated with mouse monoclonal antibodies against elastin (BA4 at 1∶100 dilution; Abcam, Cambridge, USA) or with rabbit polyclonal antibodies against collagen type X (ab58632 at 1∶150 dilution; Abcam) all diluted in 1% BSA (in PBS, pH 7.4) overnight at 4°C. Following primary antibody incubation, sections were rinsed in PBS (pH 7.4), and incubated with Texas Red (elastin) or FITC (collagen X) labeled anti-mouse or anti-rabbit secondary antibodies (1∶200 dilution; Abcam) for 2 hours at room temperature. Sections were counterstained and mounted with DAPI (Vector Laboratories) and examined by fluorescent microscopy using a Zeiss Axio-Image M1 microscope with Axiovision software (Carl Zeiss, Oberkochen, Germany).

For all immunohistochemical studies, non-specific staining was assessed by replacement of the primary antibody with non-immune serum. These experiments were completed at least three times with no positive staining detected in the negative controls.

### Transmission Electron Microscopy (TEM)

Engineered constructs and native auricular cartilage tissue samples were fixed in a Karnovsky solution for 2 hours at room temperature. Samples were then post-fixed in 1% osmium tetroxide for 1 hour at 4°C, dehydrated in graded ethanol solutions, and embedded in epoxy resin Epon 812 (Electron Microscope Science, Hatfield, PA). Ultrathin sections (70 nm) were cut and collected on copper grids, stained with uranyl acetate and lead citrate and examined using a LEO 906 transmission electron microscope (LEO Elektronenmikroskopie GmbH, Oberkochen, Germany).

### Biochemical Quantification of Matrix Constituents

Engineered construct samples were weighed again to determine the percentage sample mass of the entire developed construct. The dry weight of the engineered construct as well as native auricular cartilage tissues samples was then determined after overnight lyophilization. Samples were then digested by papain (bioreactor samples: 40 µg/mL; native tissue samples: 80 µg/mL in 20 mM ammonium acetate, 1 mM EDTA and 2 mM dithiothreitol) for 72 hours at 65°C and stored at −20°C until analysis. Aliquots of the digest were assayed separately for DNA, proteoglycan and collagen content. The DNA assay was estimated using Hoechst 33258 dye (Sigma-Aldrich) [30]. The proteoglycan content was estimated by quantifying the amount of sulphated glycosaminoglycans using 1,9-dimethylmethylene blue (DMMB) dye binding assay (Sigma-Aldrich) [31], [32] Total collagen content was determined by the determination of hydroxiproline content. Briefly, aliquots of the papain digest were hydrolyzed in 6 N HCl for 18 hours at 110°C and the hydroxyproline content was determined in the hydrolyzate using chloramine-T/Ehrlich's reagent assay [33]. Total collagen content was estimated assuming hydroxyproline accounts for 10% of the total collagen mass in cartilage [34].

### Creation of Ear Mold Constructs

To explore the possibility of creating elastic cartilaginous constructs in the shape of the external ear, rapid-prototyped tissue culture molds were created from a previously generated 3D polygonal external ear model developed from CT imaging (TurboSquid, New Orleans, LA). Surface geometry of the external ear was extracted from the 3D model (1/2 size model; 27 mm in length measured from helix to lobe) and incorporated into a cylindrical mold (sized to fit within the reactor well) fabricated out of non-cytotoxic, acrylonitrile butadiene styrene (ABS) thermoplastic by rapid-prototyping (Dimension sst 1200es, Stratasys, Eden Prairie, USA). Mold surfaces were first coated with chicken type II collagen (Sigma-Alrich) [25] and then directly seeded with ∼20,000 isolated chondrocytes (13,000 cells/cm^2^) and maintained under no-flow conditions for 48 hours. Mold cultures were grown under a constant media flow (10 µL/min) for 4 weeks with either additional IGF-1 or insulin, as described previously. Previous work has demonstrated no adverse effects of the mold material or collagen coating on the developed cartilaginous tissues [25]. After harvest, the surface morphology of both the construct and mold were measured by 3D laser scanning (SG2 Series, ShapeGrabber, Ottawa, Canada) with a resolution of 100 µm. Prior to laser scanning, the anatomically-shaped constructs were first coated with hair-spray to create a reflective surface and avoid potential issues with excess moisture on the construct surface. Shape fidelity of the engineered defect-specific construct was evaluated by determining the root-mean-square (RMS) error between the mold and construct surfaces using an iterative closest point matching algorithm [35].

### Statistical Analyses

Two separate batches of cells were used to create the engineered constructs (N = 4–6 animal donors in total) and the results of the replicate samples were pooled together prior to conducting the statistical analyses (total n = 5–8 samples/group). Due to the relatively small number of replicate samples generated from each animal, the potential effect of animal donor was not included in the statistical models. All results were expressed as the mean ± standard error of the mean (SEM). Biochemical quantification data was analyzed using a one-way ANOVA and Tukey's post-hoc testing. Data was checked prior to performing statistical tests for both normality and equal-variance. Statistical tests were conducted using statistical software (SPSS version 16, SPSS Inc., Chicago, USA) and significance was associated with *p*-values less than 0.05.

## Results

### Structure of Engineered Elastic Cartilage Constructs

**Figure 1:**
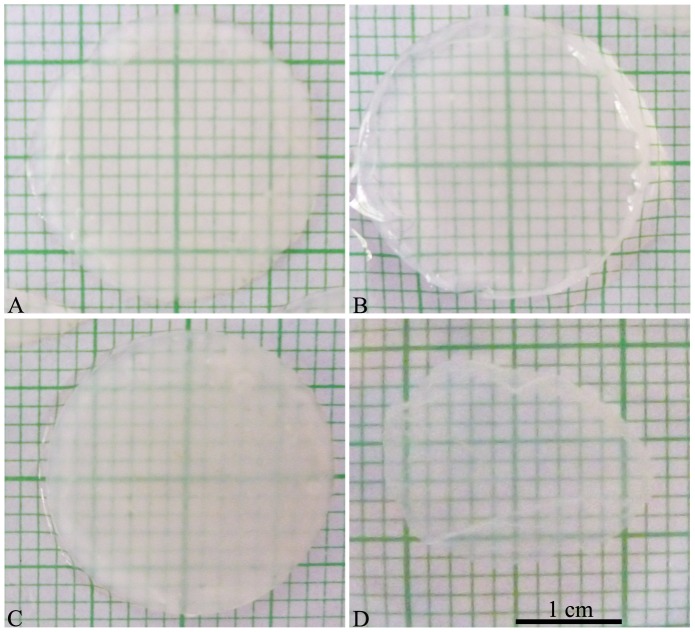
Macroscopic appearance of generated scaffold-free, engineered tissue constructs. Macroscopic appearance of the tissue generated from the monolayer cell preparations after 4 weeks of bioreactor culture. (A) Control, (B) IGF-1, (C) Insulin, and (D) TGF-β1. Scale bar: 1 cm.

Elastic cartilaginous cartilage constructs were generated from a small population of isolated cells and long-term bioreactor culture (4 weeks) using media supplemented with anabolic factors (IGF-1, insulin or TGF-β1) to improve overall tissue growth and to better localize extracellular (ECM) macromolecules within the developed tissue constructs. While each group resulted in the formation of large tissue constructs (∼3 cm^2^), cells grown in the presence of TGF-β1 did not appear to form good quality tissue constructs and were very fragile (Figure 1). Alternatively, cells grown in the presence of IGF-1 or insulin were able to form tissue constructs that were glistening white in color, firm in consistency, and easily handled. These tissue constructs were also visually thicker than the constructs grown in the presence of TGF-β1 or controls (without growth factor stimulation).

**Figure 2:**
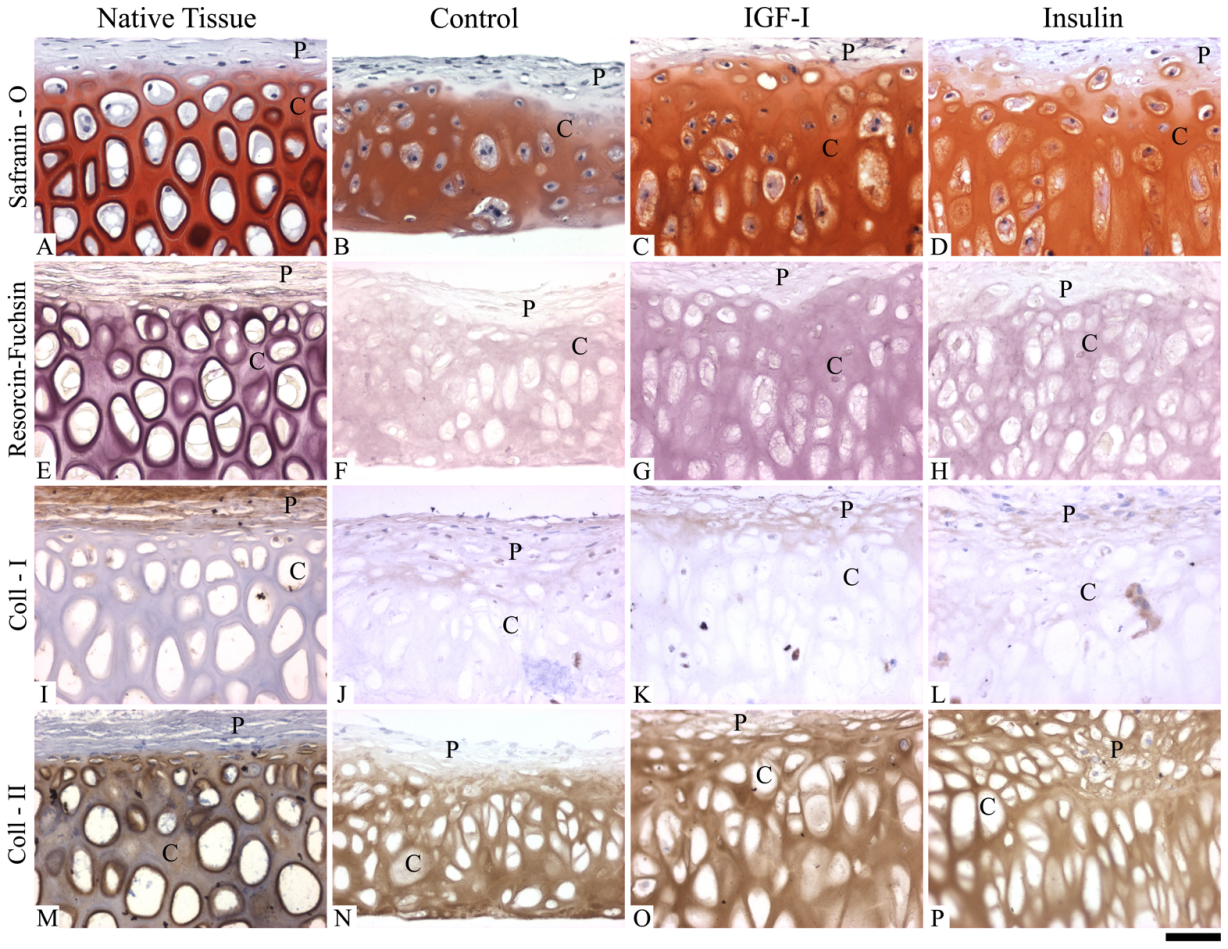
Histological and immunohistochemical appearance of generated scaffold-free, engineered tissue constructs. Histological and immunohistochemical appearance of the tissue generated from the monolayer cell preparations after 4 weeks of bioreactor culture compared to native auricular cartilage. Native auricular cartilage (A, E, I, and M), Control (B, F, J, N), IGF-1 (C, G, K, O), and Insulin (D, H, L, P). Safranin-O (sulphated proteoglycan stain) (A, B, C, D), Resorcin-Fucsin (elastin stain) (E, F, G, H), Collagen I (I, J, K, L), and Collagen II (M, N, O, P). Letters “P” and “C” refer to the perichondrial-like and cartilaginous regions, respectively. Scale bar: 30 µm.

Histological evaluation of the engineered constructs also revealed that both IGF-1 and insulin supplemented media resulted in the formation of neotissues with greater similarities to native auricular cartilage (Figure 2). While the tissue constructs (controls, IGF-1 and insulin) displayed distinct cartilaginous and perichondrial-like regions, these regions were more defined and better organized as a result of IGF-1 (Figure 2C, G, K, O) or insulin (Figure 2D, H, L, P) supplementation. Within the cartilaginous region, control cultures (without growth factor supplementation) positively stained for sulphated proteoglycans (safranin-O) (Figure 2B) and elastin (resorcin-fuchsin) (Figure 2F) with round and oval shaped chondrocytes and some binucleated cells. IGF-1 and insulin treated cultures displayed more intense staining for both sulphated proteoglycans (Figure 2C, D respectively) and elastin (Figure 2G, H respectively) with enlarged binucleated cells with elongated morphologies more similar to native cartilage (Figure 2A, B respectively). The developed perichondrial-like region was similar amongst the groups, which did not stain for sulphated proteoglycans (Figure 2A) and contained smaller cells similar to fibroblasts. In contrast, TGF-β1 supplemented cultures appeared to resemble poorly organized tissue without the presence of hypertrophic cells or a distinct perichondium-like region (). For this reason, further immunohistochemical assessment on the TGF-β1 cultures were not undertaken.

**Figure 3:**
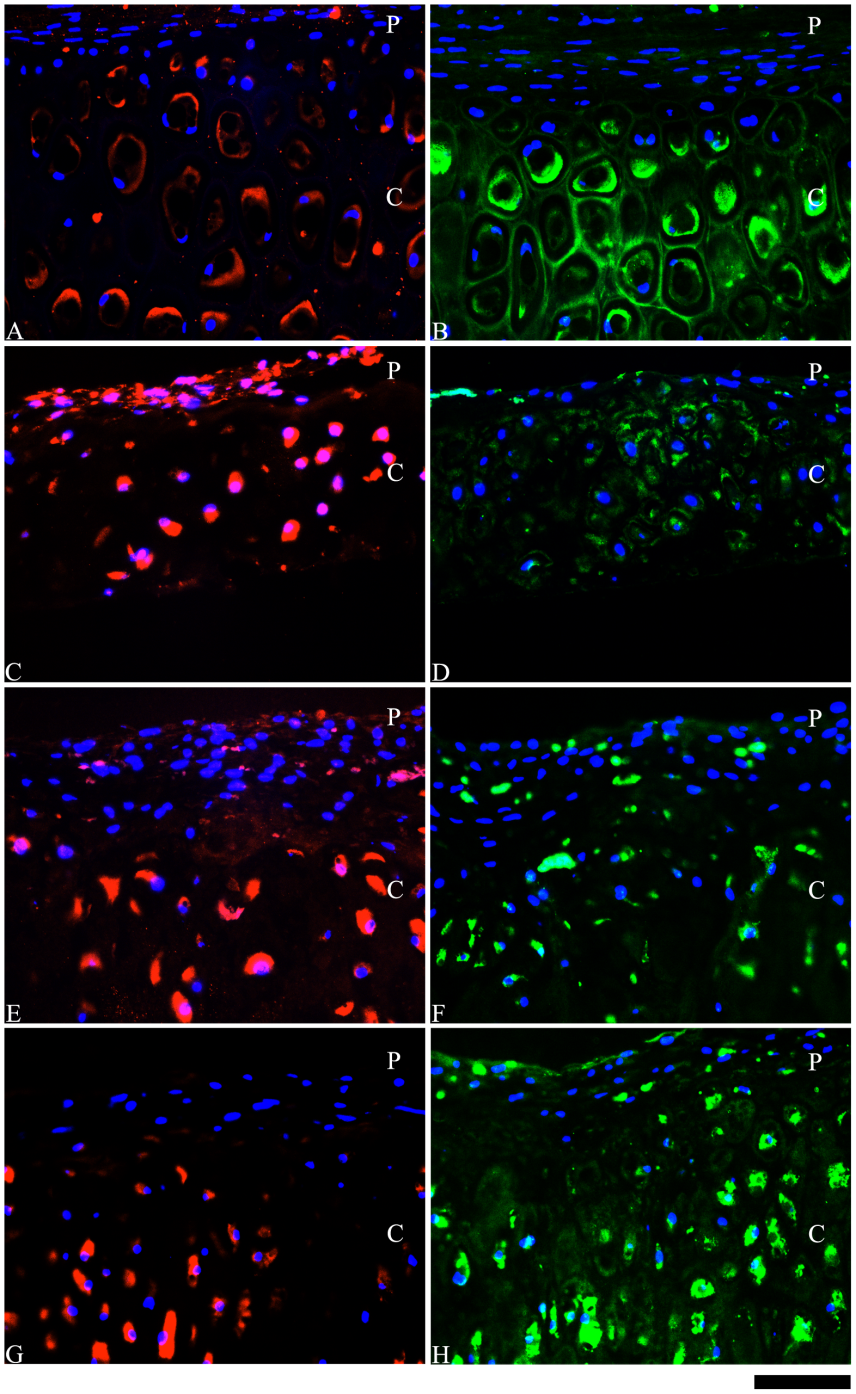
Immunohistochemical localization of elastin and collagen X in the scaffold-free, engineered tissue constructs. Immunohistochemical localization of elastin (red) (A, C, E, G) and collagen X (green) (B, D, F, H) in the engineered elastic cartilaginous constructs generated from monolayer preparations after 4 weeks of bioreactor culture compared to native auricular cartilage. Native auricular cartilage (A, B), Control (C, D), IGF-1 (E, F), and Insulin (G, H). Letters “P” and “C” refer to the perichondrial-like and cartilaginous regions, respectively. Scale bar: 30 µm.

Immunohistochemical assessment showed that collagen I was primarily located in the perichondium-like region within the developed constructs (controls, IGF-1 and insulin) (Figure 2J–L); however, the insulin supplemented cultures displayed few cells with intracellular staining for collagen I (Figure 2L) in the cartilaginous region. The cartilaginous regions stained positive for both collagen II (Figure 2N–P) and collagen X (Figure 3D, F, H) in all of the developed constructs. Collagen X staining appeared to be more intense in the insulin supplemented cultures (Figure 3H) compared to the cultures supplemented with IGF-1 (Figure 3F) or the control cultures (Figure 3D) (no growth factor supplementation). However, in each of these constructs, sparse staining for collagen X staining was also detected in the perichondrial-like region. The presence of elastin was observed in the engineered constructs, which was also affected by growth factor supplementation (Figure 3C, E, G). Without growth factor supplementation (controls), elastin was primarily observed intracellularly and was present in both the cartilaginous and perichondrial-like regions (Figure 3C). However, supplementation with either IGF-1 (Figure 3E) or insulin (Figure 3G) appear to be better localize elastin expression in the cartilaginous region, with a greater effect observed in response to insulin supplementation. Alternatively, in response to IGF-1 supplementation, elastin expression was observed both intracellularly and in the developed ECM (Figure 3E).

### Tissue Ultrastructure

**Figure 4:**
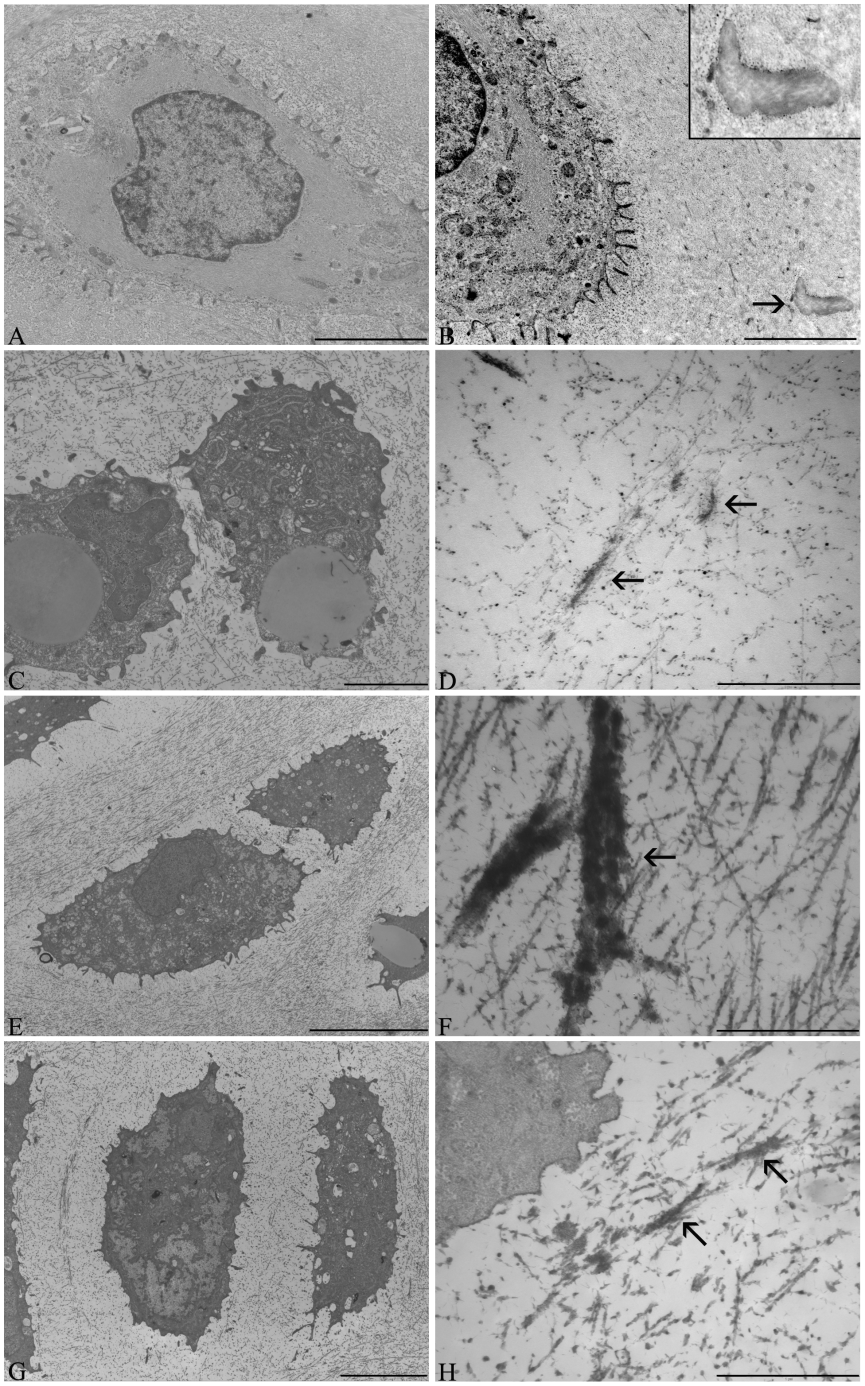
Transmission microscopy of cellular ultrastructural and extracellular matrix organization. Transmission electron micrographs, within the cartilaginous region, of the engineered elastic cartilaginous constructs after 4 weeks of bioreactor culture compared to native auricular cartilage. Native auricular cartilage (A, B), Control (C, D), IGF-1 (E, F), and Insulin (G, H). Separate images were taken for both the cellular ultrastructure (A, C, E, G) and organization of the extracellular matrix (B, D, F, H). Arrows denote presence of elastic fibres. In native auricular cartilage, the elastic fibres (B and insert) appear to be more organized, dense and weakly contrasted compared to the elastic fibres present in the engineered constructs (D, F, H). Scale bars: A, C, E, G: 5 µm; B: 2 µm; D, F, H: 1 µm.

Transmission electron microscopy (TEM) evaluation of tissue ultrastructure showed that the cells within the engineered tissue constructs resembled chondrocytes and the developed tissue appeared to display all of the characteristics of native auricular cartilage (cytoskeleton, lipid droplets, defined territorial and interterritorial matrices) (Figure 4). Extracellular elastin, observed as electron-dense amorphous aggregates, was also detected in all of the engineered constructs (Figure 4D, F, H). The control cultures (Figure 4D) (no growth factor supplementation) displayed sparse and thin elastic fibres, which were increased in response to both IGF-1 (Figure 4F) and insulin (Figure 4H) supplementation. Similar to the immunohistochemical results, the most prominent and thickest elastic fibres were detected in the ECM of the IGF-1 supplemented cultures (Figure 4F). However, compared to native auricular cartilage (Figure 4B), the elastic fibres in the engineered tissues appeared to be less dense and organized.

### Physical and Biochemical Properties of Accumulated Extracellular Matrix

Growth factor supplementation also had an observable effect on biochemical and physical properties of the engineered elastic tissue constructs in terms of tissue cellularity, ECM accumulation, thickness, and resultant mechanical properties. Although no statistical differences were observed between IGF-1 and insulin supplementation, these cultures had significantly higher DNA (83–93% increase, *p*<0.01), proteoglycan (3.2–3.3 fold increase, *p*<0.01), and collagen (2.1–2.5 fold increase, *p*<0.01) contents compared to the controls (no growth factor supplementation) (Table 1). Similarly, construct mass (wet and dry weights) were also significantly higher in the IGF-1 and insulin supplemented cultures (1.9–2.3 fold increase, *p*<0.01) without corresponding changes in water content (Table 1). When normalized to tissue weight or cellularity, generally similar effects of IGF-1 and insulin supplementation were observed, with the exception of tissue cellularity, which was similar amongst all of the cultures (Table 2). However, when compared to native auricular cartilage, collagen accumulation and cellularity were lower (*p*<0.05) in the engineered constructs. Proteoglycan accumulation approached the values of native cartilage without growth factor supplementation (controls), whereas was higher (*p*<0.05) in the constructs stimulated by wither IGF-1 or insulin (Table 2). Similar to the previous results, supplementation with TGF-β1 resulted in the generation of constructs with significantly less ECM and lower cellularity compared to all of the other developed constructs (controls, IGF-1 or insulin) (Tables 1 and 2).

**Table 1:** Biochemical properties of scaffold-free, engineered tissues.

	Control	TGF-β1 [0.1 nM]	IGF-1 [10 nM]	Insulin [100 nM]
	(n = 7)	(n = 5)	(n = 8)	(n = 8)
Total DNA (µg/construct)	30±4^a^	4.4±0.4^a^	58±6	55±6
Total PG (µg/construct)	1918±374^a^	95±42^a^	6258±880	6356±1071
Total Collagen (µg/construct)	2345±903^a^	504±137^a^	5777±1780	4938±1562
Wet Weight (mg)	87±13^a^	23±5^a^	197±20	183±14
Dry Weight (mg)	9±1^a^	1.9±0.4^a^	17±1	18.2±0.8
Water Content (%)	89.6±0.5	91.9±0.4	91.1±0.2	90.6±0.2

Biochemical Properties of Engineered Elastic Cartilaginous Tissues. Data presented as mean ±SEM.

PG: Proteoglycans.

a Significantly different from all other groups (*p*<0.01).

**Table 2:** Biochemical properties of scaffold-free, engineered tissues compared to native auricular cartilage.

	Control	TGF-β1 [0.1 nM]	IGF-1 [10 nM]	Insulin [100 nM]	Native Tissue
	(n = 7)	(n = 5)	(n = 8)	(n = 8)	(n = 6)
DNA [µg/mg dry wt.]	3.4±0.3	2.7±0.5	3.3±0.1	3.1±0.1	2.3±0.2^a^
PG [µg/mg dry wt.]	219±39	44±13^a^	362±34	359±40	231±26
Collagen [µg/mg dry wt.]	163±28	263±17	332±87^b^	264±73	496±35^a^
PG/DNA [µg/µg]	70±18^a^	20±8^a^	111±12	114±11	133±14
Collagen/DNA [µg/µg]	68±23	110±23	101±27	80±22	230±30^a^
Collagen/PG [µg/µg]	1.1±0.2	7±2^a^	0.8±0.1	0.6±0.1^a^	2.2±0.2^a^

Biochemical properties of engineered elastic cartilaginous tissue and native auricular tissue normalized to dry weight or DNA content. Data presented as mean ±SEM.

PG: Proteoglycans.

a Significantly different from all other groups (*p*<0.05).

b Significantly different from control (*p*<0.05).

The thickness of the cultures was significantly increased with IGF-1 and insulin supplementation (2.8-fold increase, *p*<0.01) and were similar to that of native auricular cartilage (Table 3). While the elastic modulus of the constructs was improved by growth factor supplementation (54–69% increase, *p* = 0.07), the engineered constructs displayed lower moduli when compared to native auricular cartilage (Table 3). However, the IGF-1 and insulin supplemented constructs appeared to possess a similar Poisson's ration to that of native cartilage (Table 3). Note that as the TGF-β1 constructs were too fragile, it was not possible to reliably determine tissue thickness or mechanical properties.

**Table 3:** Physical properties of properties of scaffold-free, engineered tissues.

	Control	IGF-1 [10 nM]	Insulin [100 nM]	Native Tissue
Thickness [µm]	168±17^a^	463±50	470±52	506±18
Elastic Modulus [kPa]	13±4	22±2^b^	20±2^b^	100±19^a^
Poisson's Ratio	0.31±0. 01	0.29±0.01	0.29±0.01	0.28±0.02

Thickness and mechanical properties of engineered elastic cartilaginous tissues. Data presented as mean ±SEM; *n* = 7–8/group.

a Significantly different from all other groups (*p*<0.01).

b Trend from control (*p* = 0.07).

### Development of Anatomically Shaped, Scaffold-Free Auricular Cartilage Constructs

**Figure 5:**
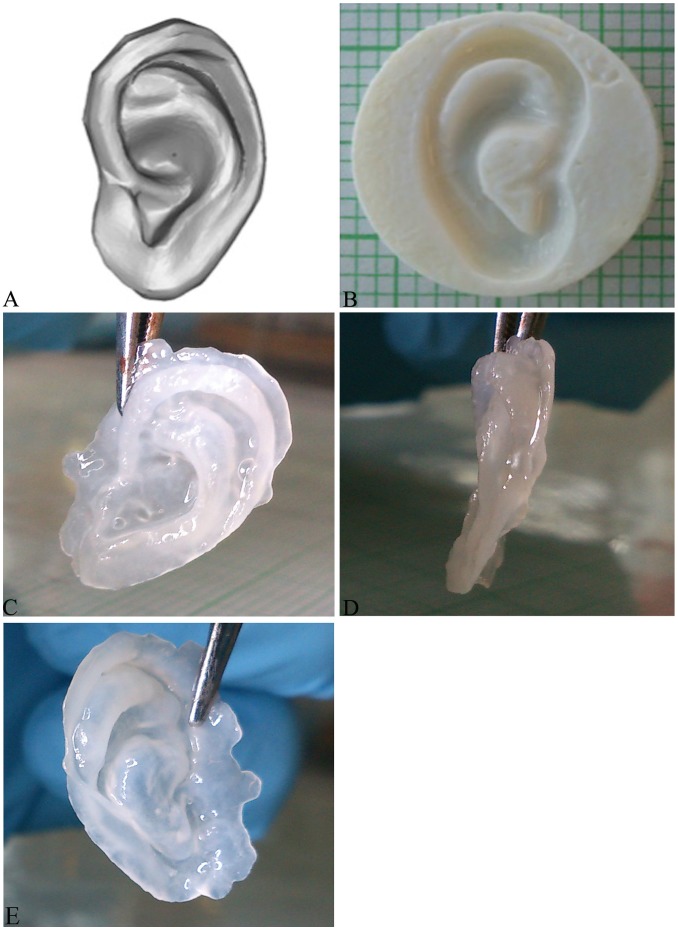
Development of 3D shaped scaffold-free, engineered auricular cartilage constructs. Macroscopic images of 3D ear model (1/2 size model; 27 mm in length measured from helix to lobe) (TurboSquid) (A) and corresponding rapid-prototyped (negative) tissue culture mold (B). Resultant scaffold-free tissue engineered auricle after 4 weeks of bioreactor culture: IGF-1 side view (C), IGF-1 frontal view (D), and Insulin side view (E).

Due to the promising effects of IGF-1 and insulin supplementation of the developed elastic cartilaginous tissue constructs, isolated cells were seeded in rapid-prototyped tissue culture molds in the shape of the external ear. After 4 weeks of the bioreactor culture, resultant tissue constructs could be easily removed from the mold, were flexible and appeared to retain their 3D shape (Figure 5). 3D laser scanning of the construct and rapid-prototyped molds were then conducted to assess the shape fidelity. Construct shape fidelity appeared to be preserved with very good agreement between the mold and engineered construct. The average positional error (RMS) between the construct and corresponding mold surfaces were 368±20 µm (*IGF-1*, n = 4) and 391±36 µm (*insulin*, n = 4), with the majority of the construct surface within 100 µm (lower than the resolution of the 3D scanner) of the mold and higher deviation noted primarily at regions of high curvature (Figure 5).

## Discussion

Sculpted autologous costal cartilage grafts for the reconstruction of the external ear have good long-term durability [36]–[38] as well as the potential to grow with the patient [1], [3]. First introduced by Tanzer [38], this multi-stage procedure (e.g. 4-stage Brent procedure [36] or 2-stage Nagata procedure [37]) is recognized as surgically demanding and involves harvesting of a large amount of autologous donor cartilage for subsequent shaping. This can result in significant donor-site morbidity and patients must have a sufficient supply of donor cartilage to be candidates — potentially delaying the age at which the reconstruction can be performed [1], [3]. To address this concern, we have developed an approach to generate scaffold-free engineered auricular cartilage constructs directly from a small amount of isolated cells [13] without the need for additional cell expansion. Although the bioreactor cultivated constructs appeared to adopt the structural appearance of native auricular cartilage, the constructs displayed limited expression and poor localization of elastin (a fundamental constituent of the elastic cartilage matrix). In the present study, the effect of growth factor supplementation (insulin, IGF-1, or TGF-β1) was investigated to stimulate elastogenesis as well as to improve overall tissue formation. While previous studies have shown that these factors can upregulate elastin expression [18]–[21], stimulation with TGF-β1 did not elicit positive effects on tissue formation in bioreactor culture. This result may be due to the observed differences between transient as opposed to continuous exposure to growth factors in culture. Ng *et al.* [39] demonstrated that transient exposure (14 of 42 days) of articular chondrocytes to TGF-β1 or TGF-β3 resulted in improved cartilaginous tissue formation and mechanical properties as opposed to continuous exposure for the same period of time. Similarly, Arévalo-Silva *et al.* [40] observed that 3 weeks of continuous exposure of auricular chondrocytes to TGF-β1 also resulted in poor quality tissue formation. These effects have been attributed to the temporal regulation of growth factors (including TGF-β1) *in vivo* during both cartilage development and wound healing [41], [42].

In contrast to the effects of TGF-β1, a significant improvement in growth and properties of the engineered elastic cartilage constructs were observed in response to supplementation with either insulin or IGF-1. Engineered constructs grown in the presence of insulin or IGF-1 displayed thicknesses approaching native auricular cartilage with improved deposition of cartilaginous ECM and resultant mechanical properties. IGF-1 has long been established as a potent anabolic factor used to improve the formation of engineered cartilage, with several studies showing increased deposition of cartilaginous ECM and improved mechanical properties of the developed constructs [22], [23], [43]. Similarly, while insulin-transferrin-selenium (ITS) supplemented media has also been shown to improve cartilage tissue formation *in vitro* [44], [45], recent work has shown that insulin alone is most likely responsible for these effects [46], [47]. Insulin and IGF-1 belong to the same peptide family and the similarity between their molecular structures is probably responsible to similar effects observed [48]. This is in agreement with our findings showing increased elastin expression (by IHC) and better elastic fibre formation (by TEM) in response to long-term stimulation by either peptide. Potentially attributable to the concentrations used, supplementation with IGF-1 resulted in apparently more mature elastic fibre organization compared to supplementation with insulin. Interestingly, supplementation with insulin or IGF-1 also served to better localize elastin expression within the cartilaginous region of the developed constructs. While all of the developed constructs possessed distinct perichondrial and cartilaginous regions (with the exception of constructs supplemented with TGF-β1), control constructs (no growth factor supplementation) expressed elastin (primarily intracellularly) in both the cartilaginous and perichondrial-like regions. However, when grown in the presence of insulin or IGF-1, elastin expression in the perichondrial-like region was reduced (insulin) or virtually undetected (IGF-1), similar to the absence of elastin expression within perichondrium of native auricular cartilage. While the underlying mechanisms responsible for this effect are currently unknown, previous studies suggest that the response of perichondrial cells to exogenous insulin and IGF-1 may be highly regulated. During development, perichondrial cells (compared to chondrocytes) express a higher proportion of IGF receptors (IGF-1R) [49], [50] and IGF binding proteins (IGFBP-4, IGFBP-5) [50]. The perichondrium also contains a population of chondrogenic progenitors (chondroblasts) [51], [52], which have been shown to differentiate into chondrocytes in response to exogenous IGF-1 [52]. Lastly, the expression of collagen X was also significantly enhanced in response to stimulation by these peptides. Collagen X is known as a marker of chondrocyte hypertrophy and mineralizing cartilages [6], [53], but is also expressed in non-mineralizing tissues, including auricular and nasoseptal cartilages [6]. Previous studies have shown that IGF-1 can promote chondrocyte hypertrophy [54], [55] and our results suggest that insulin potentially has a similar effect as the changes in collagen X expression and cellular morphology were quite similar in response to either insulin or IGF-1 supplementation.

To determine whether anatomically shaped scaffold-free auricular cartilage constructs could be developed, isolated cell were grown in rapid prototyped culture molds. Resultant tissue constructs had good agreement with the original cell culture mold (average RMS error between construct and mold of less than 400 µm). This result was similar to our previous work in generating shaped scaffold-free articular cartilage constructs that could be matched to the defect site [25]. However, using this approach, stronger shape correlations between the construct and original mold were observed with articular chondrocytes (<120 µm average positional error). While this effect may be due to the differences in cell types, the external ear has a more complex shape compared to the curvature(s) of cartilage within the knee. On this note, the recreation of predominant over-hanging features of the auricular (e.g. crus of the helix, tragus, and anti-tragus) for certain individuals might prove to be challenging and may require a modular mold to ensure accurate 3D shaping of the developed constructs. Numerous studies have also demonstrated that engineered auricular cartilage constructs can be developed in the shape of the external ear with generally good spatial agreement to the desired 3D shape [1], [3], [11], [12], [56], [57]. However, shape fidelity in auricular cartilage tissue engineering is a concern as prior studies have shown that the engineered scaffolds can be susceptible to significant shrinkage and/or shape distortions after subcutaneous implantation [58]. Thus, while our initial results appear promising, the next steps are to further characterize the anatomically-shaped constructs (mechanically, histologically, biochemically) and then determine whether these scaffold-free constructs can maintain their 3D shape after implantation.

In this study, we have investigated the potential of growth factor stimulation during bioreactor culture to improve expression and localization of cartilaginous ECM proteins in scaffold-free auricular cartilage constructs. Media supplementation with either insulin or IGF-1 was successful at improving overall tissue growth and properties with the developed constructs obtaining the thickness native auricular cartilage. Insulin and IGF-1 also served to upregulate elastogenesis and better localize elastin expression to the cartilaginous regions of the developed constructs in a similar fashion to native cartilage. In contrast, supplementation with TGF-β1 had appeared to have a negative effect on tissue formation and resultant constructs were generally thin and fragile. These results also suggest that there may be some added benefit to the use of a combination of insulin and IGF-1 on the formation and properties of engineered auricular cartilage constructs. In addition, other combinations of growth (including TGF-β1) should also be investigated, as previous work has shown that the combined effect of insulin and TGF-β1 serves to upregulate elastic fibre formation in smooth muscles cell seeded fibrin constructs [20]. Scaffold-free auricular cartilage constructs could also be formed in the shape of the external ear using rapid prototyped 3D culture molds. Although very good spatial agreement between the developed construct and mold was achieved, future studies will be aimed assessing potential changes in construct shape and properties after subcutaneous implantation and translation to the development of engineered human auricular cartilage constructs.

## Supporting Information

Figure S1 **Histological appearance engineered tissue constructs supplemented with TGF-β1.** Histological appearance of the tissue generated from the monolayer cell preparations after 4 weeks of bioreactor culture supplemented with TGF-β1 stained with hematoxylin & eosin (H&E; general connective tissue stain). Note that supplementation with TGF-β1 resulted in tissue constructs were poorly organized without the presence of hypertrophic cells or a distinct perichondium-like region. Scale bar: 30 µm.
(TIFF)
