# Supplementary figures and images for: Growth Factor Stimulation Improves the Structure and Properties of Scaffold-Free Engineered Auricular Cartilage Constructs
Source: PLoS One. 2014 Aug 15;9(8):e105170. doi: 10.1371/journal.pone.0105170 (PMC4134285; doi:10.1371/journal.pone.0105170)

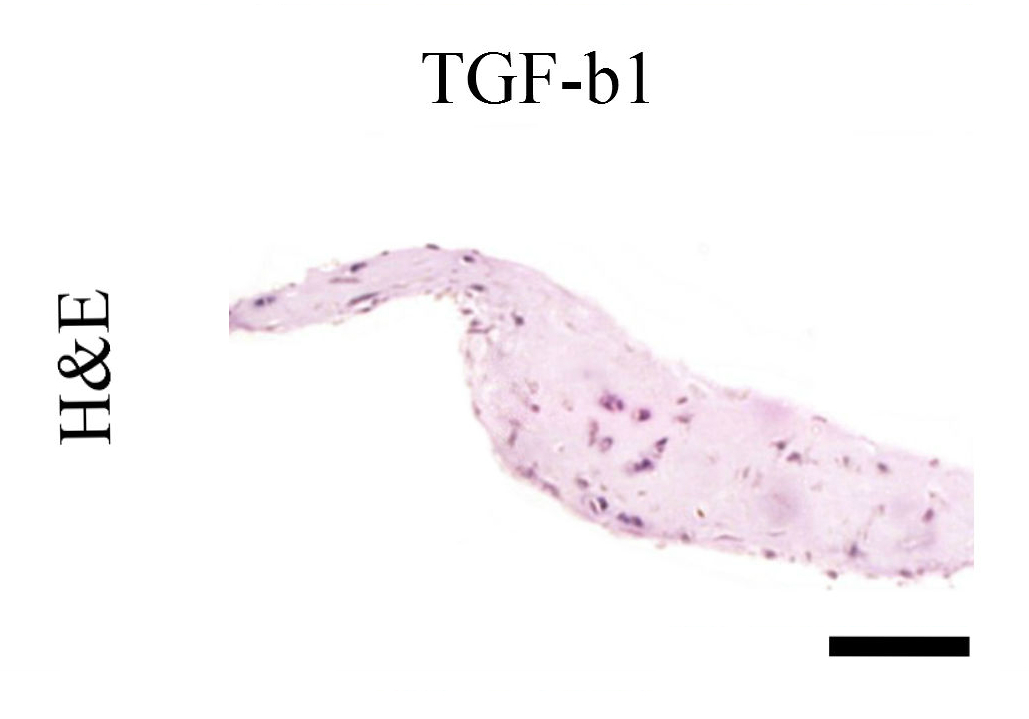

Supplement: Figure S1 — Histological appearance engineered tissue constructs supplemented with TGF-β1. Histological appearance of the tissue generated from the monolayer cell preparations after 4 weeks of bioreactor culture supplemented with TGF-β1 stained with hematoxylin & eosin (H&E; general connective tissue stain). Note that supplementation with TGF-β1 resulted in tissue constructs were poorly organized without the presence of hypertrophic cells or a distinct perichondium-like region. Scale bar: 30 µm. (TIFF) [file pone.0105170.s001.tiff]
